# Effects of Mindfulness-Based Cognitive Therapy for Chronic Pain: A Multicenter Study

**DOI:** 10.3390/ijerph18136951

**Published:** 2021-06-29

**Authors:** Estela María Pardos-Gascón, Lucas Narambuena, César Leal-Costa, Antonio Jesús Ramos-Morcillo, María Ruzafa-Martínez, Carlos J. van-der Hofstadt Román

**Affiliations:** 1 Sexual and Reproductive Health Unit, Novelda Health Center, 03660 Alicante, Spain; estela.maria.pardos.gascon@gmail.com; 2 Child-Youth Mental Health Unit, Can Misses Hospital, 07800 Ibiza, Spain; narambuenalucas@gmail.com; 3 Faculty of Nursing, University of Murcia, 30100 Murcia, Spain; maruzafa@um.es; 4 Hospital Psychology Unit, Department of Health Psychology, Institute of Health and Biomedical Research of Alicante (ISABIAL), General University Hospital of Alicante, Miguel Hernández University, 03010 Alicante, Spain; cjvander@umh.es

**Keywords:** chronic pain, MBCT, multicenter study, self-efficacy, sleep, pain intensity, quality of life, depression

## Abstract

The prevalence of chronic pain in Spain is 15%. The objective of this study was to evaluate the efficacy of mindfulness-based cognitive therapy on patients with chronic pain. A quasi-experimental design of repeated measures pre- and post-test (*N* = 57) was carried out at three hospitals from the province of Alicante. Self-reported assessment measurements of pain intensity, anxiety-depression symptoms, perception of health status, interference of pain on sleep, self-efficacy in pain, acceptance, and mindfulness attitude were included. The T-test indicates significant differences in intensity of present pain, mental quality of life, and depression (medium effect sizes), as well as in self-efficacy: total score, symptom management and pain control (medium effect sizes), sleep disturbances and quantity of sleep (large effect sizes). MBCT is effective in reducing many symptoms in patients with chronic pain, although its maintenance needs to be further investigated.

## 1. Introduction

Non-oncological chronic pain is defined as pain that lasts longer than three months after an injury, felt for longer after the injury or even without it, and which is not caused by an oncological process [1]. This type of pain is one of the 10 most prevalent medical conditions in the world and causes the longest period of disability [2]. Specifically, there is a prevalence of 19% in Europe [3] and 15% in Spain [4]. Economically, chronic pain implies costs between 2% and 2.8% of the gross domestic product in Spain.

The experience of pain is highly subjective and has a wide-ranging inter-individual variability, as first shown by Melzack and Wall [5] and Melzack [6]. Its response to medical treatment is partial, and it is currently accepted that psychological and emotional processes exert a modulating function in the sensorial signal of pain [7], which justifies research on this subject [8].

Presently, the psychological treatment of choice is cognitive behavioral treatment (CBT) [9,10,11], and the literature points to improvements in pain, disability, and mood with its use and has also generally demonstrated good tolerability, acceptability, and efficacy for chronic pain conditions and migraines in children [12]. Although this treatment has been studied in a limited number of pathologies, its mechanisms are not clear and its effect size tends to be small [1]. Likewise, some authors [13] suggested that these effects are due to broader adaptive changes that occur during the coping process, instead of variables that are specifically cognitive.

In the last few years, a strong interest has appeared for the treatments based on mindfulness for chronic pain. These treatments are based on the acceptance of pain, the decrease in avoidance, the de-identification with mental processes, and the reduction of the hyper-alert state towards the feelings of pain [14]. The meta-analyses generally agree on their effectiveness [15,16,17,18,19,20] and some authors believe these treatments may even last longer than CBT in the medium term [21].

Within these treatments, Day [22] has developed the mindfulness-based cognitive therapy for chronic pain (MBCT-CP). This was a little-studied approach until now, although it is one of the most specific for this type of pathology. The results have been shown to be promising for the reduction of the interference due to pain and the increase in self-efficacy and acceptance of pain, with size effects that are similar to other interventions of choice [22,23].

Therefore, we hypothesize that an intervention based on MBCT-CP will have a positive impact on levels of pain, anxiety, depression, self-efficacy, quality of life, and sleep attributes.

The objective of the present study was to evaluate the efficacy of the MBCT for chronic pain in a sample of adult patients with different medical diagnoses associated with chronic pain in the province of Alicante, Spain. The specific objectives were to evaluate the effectiveness of said program based on levels of pain, anxiety, depression, self-efficacy, quality of life, and sleep attributes.

## 2. Materials and Methods

### 2.1. Design

Quasi-experimental design with pre- and post-test repeated measures, with an intragroup comparison of measurement before and after the intervention.

### 2.2. Participants

Eligible participants included a sample of adult patients with different medical diagnoses associated with chronic pain in the province of Alicante, Spain. To be included in the study, the participants: (1) had to be older than 20 years old; (2) had non-oncological pain lasting longer than 3 months, with a moderate-severe intensity, as diagnosed by the Unit of Pain doctors; (3) had pain that interfered in at least one area of everyday life; (4) had to be self-sufficient for completing the self-applied tests; and (5) had to freely accept to participate in the study by providing their written informed consent. The following subjects were excluded: (1) those who had diminished capacities for providing informed consent; and (2) those who had a severe clinical comorbidity; or (3) an acute phase psychotic disorder. The sample was obtained through a waiting list of clinical psychology from the units of pain, from June 2018 to January 2019.

A sample size of 40 achieves 100% power to detect a mean of paired differences of 5 with an estimated standard deviation of differences of 1.0 and with a significance level (alpha) of 0.05 using a two-sided paired *t*-test. Thus, for 6 groups of subjects, a total of 48 subjects would be needed. Ultimately, a sample of 57 participants was obtained through a convenient, non-randomized sample at the units of chronic pain from three hospitals located in the province of Alicante: General University Hospital of Alicante (GUHA), Marina Baixa Hospital (MBH) (Villajoyosa), and the Vega Baja Hospital (VBH) (Orihuela).

### 2.3. Instruments

A data collection booklet was created for data collection, which included the following measurements:

- Sociodemographic characteristics: age, sex, marital status, and occupation.
- Self-reported pain intensity: the pain in the last three days was assessed with the visual analogue scale (VAS) [24], with scores ranging from 0 (absence of pain) to 10 (the worst pain imaginable), with slight pain = VAS < 4, moderate between 4–6, and severe with VAS > 6. VAS has reported high test-retest reliability (ICC = 0.71–0.99), convergent validity values ranging from 0.30 to 0.95, and moderate concurrent validity (0.71–0.78) when compared with a numeric pain rating scale [25,26,27,28]. Likewise, a Likert scale with 5 points was utilized to assess the intensity of present pain, with 4 being extremely intense, 3—intense, 2—moderate, 1—mild, and 0—none.
- Anxiety-depression symptoms: the hospital anxiety and depression scale (HADS) [29] which was validated in a Spanish population with chronic pain [30] was utilized. This is a self-completed form with 14 items, with a Likert-type response scale with 4 points. The overall score oscillates between 0 and 42, with a range between 0–21 for each sub-scale. Its reliability was adequate (anxiety subscale: α = 0.81; depression subscale: α = 0.82) as it is reported in previous studies [31].
- Perception of status of health: the Spanish version [32] of the short form health survey 12 (SF-12) questionnaire was utilized [33]. It consists of 12 items taken from the SF-36, with a Likert-type response scale ranging from three to six points. The result is a measurement of overall physical and mental well-being, with scores ranging from 0 (worse state of health possible), to 100 (best state of health possible), with an adequate reliability for both scales (physical summary: α = 0.84; mental summary: α = 0.72) in line with previous research [34].
- Interference of pain on sleep: sleep was measured with the medical outcomes study sleep scale (MOS Sleep Scale) [35]. It is composed by 12 items that explore the impact of external stimuli on the attributes of the architecture of sleep (suitability, optimum sleep, quantity, abrupt awakenings, snoring, altered sleep, and somnolence). Likewise, it is used to evaluate the overall interference with sleep index with 6 or 9 items, with a range of responses from 0 (no interference or impact) to 100 (maximum interference possible). This scale obtained a reliability between 0.62 and 0.83 with Cronbach’s Alpha, as in studies with patients suffering from neuropathic pain [36].
- Perception of self-efficacy in the management of pain: this was measured with the chronic pain self-efficacy scale [37], which obtained an adequate reliability (subscale of control of symptoms: α = 0.84, subscale of physical functioning: α = 0.95; subscale of pain management: α = 0.70, total self-efficacy score: α = 0.90) as in previous research of the authors of the scale. This questionnaire was composed by 19 items with a Likert-type response scale of 10 points, with a greater score indicating a greater self-efficacy.
- Acceptance of pain: the chronic pain acceptance questionnaire (CPAQ) [38] was utilized. It includes 20 items, and a total score is obtained which evaluates the degree of acceptance of chronic pain and two subscales: engagement in activities (EA), and pain willingness (PW), both with a good reliability (EA: α = 0.80 and PW: α = 0.72) as it is reported in previous literature [39].
- Mindfulness state: measured with the mindful attention awareness scale (MAAS) [40]. This was validated with a Spanish population [41], and a good reliability was found (α = 0.88). The final score is obtained from the arithmetic mean of 15 items, and the highest scores indicate a greater state of mindfulness.

### 2.4. Procedure

The participants from the waiting list at the unit of pain were called to participate in group therapy sessions with 10 to 12 participants per group. In the first session, the questionnaires and the organization of the sessions were explained. The mindfulness-based cognitive therapy for chronic pain [22], was applied, which was composed of 8 weekly group sessions that lasted around an hour and a half (see Table 1). The intervention was performed by 6 3rd-year clinical psychology residents with experience in group therapy, but without specific training on mindfulness. Four groups were set up at the GUHA, (*n* = 23), 2 groups at the MBH (*n* = 11), and 2 groups at the VBH (*n* = 23) between September 2018, and June 2019. The research authors translated the mindfulness-based cognitive therapy for chronic pain manual [22] patient materials, and these were provided to the participants.

### 2.5. Confidentiality

The collection, treatment, and use of the data required for this study was conducted in agreement with the contents of Organic Law 15/1999 related to personal data, and its regulation for its development, in this case (Royal Decree 1720/2007); as well as Regulation 2016/679 from the European Parliament and Council, dated 27 April 2016, related to the treatment of personal data, as well as any norm and/or legislation that should be applied. Likewise, the research project was approved by the Ethics Committee for Research with drugs (CEIm) from the GUHA (Ref.CEIm: PI2018/109 Ref.ISABIAL:180296).

### 2.6. Statistical Analysis

The software IBM SPSS Statistics^®^ v.24 (IBM, Armonk, NY, USA) was utilized to analyze the descriptive statistics according to the nature of the variables, as well as the *t*-test for related samples, after verifying normality (Kolmogorov–Smirnov), and homoscedasticity (Levene’s test). Complementarily, the effect size was analyzed with Cohen’s d, considering a d between 0.2 and 0.49 as “small”, a d from 0.5 to 0.79 as “medium”, and d greater than 0.8 as “large” [42].

## 3. Results

**Figure 1:**
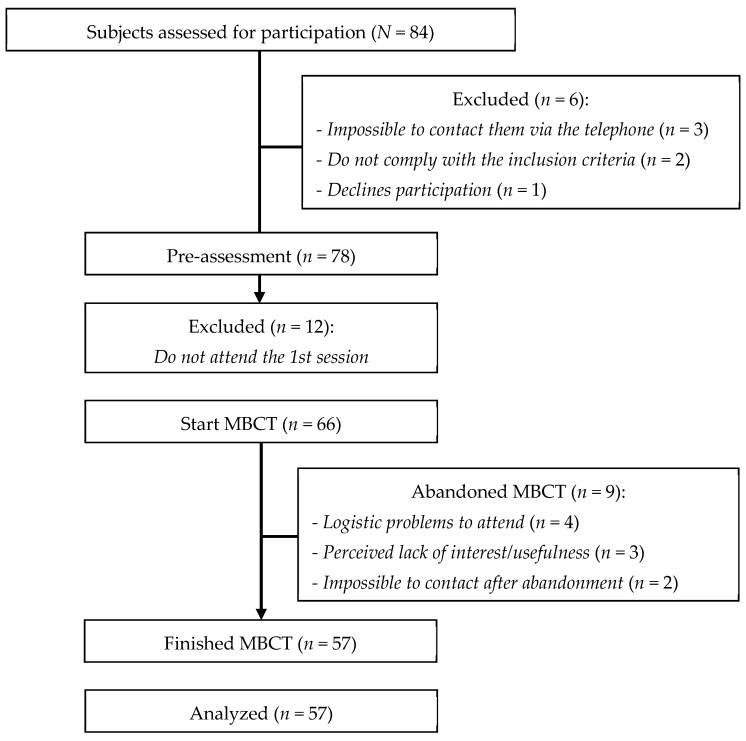
Flow diagram of the participants.

Of the 84 initial participants, 57 finished the program (response rate of 67.85%) (see Figure 1). The sample was comprised mostly by women (77.2%), aged between 32 and 79-years-old (M = 55.96), duration of illness ranged from 3 to 20 years (M = 10), married (57.9%) and retired (26.3%). Most of them (38.6%) had a diagnosis of low back pain/lumbosciatic pain, followed by neck pain or cervicobrachialgia (12.3%), fibromyalgia (12.3%), and rheumatic arthritis (10.5%), while 26.3% had a heterogeneous group of multiple diagnoses that imply chronic pain: idiopathic pain (*N* = 3), neuropathic pain (*N* = 3), failed back syndrome (*N* = 3), chronic postsurgical pain (*N* = 2) diabetes (*N* = 2), multiple sclerosis (*N* = 1), and phantom limb pain (*N* = 1). Lastly, 40.4% were GUHA patients, with the same percentage for VBH, while the MBH patients were less numerous (Table 2).

**Figure 2:**
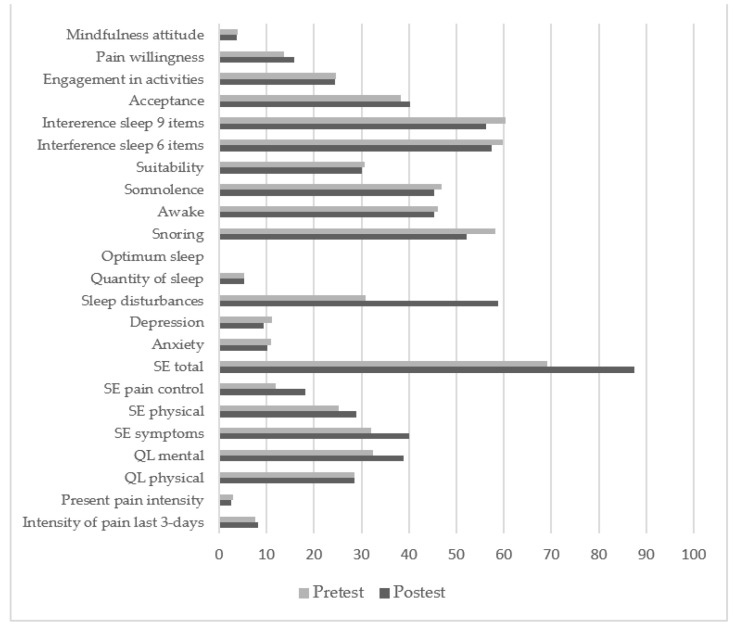
Means before and after the treatment for all the variables. QL = quality of life; SE = self-efficacy.

After verifying the homocesdasticity and normality, the results of the *t*-test (see Table 3 and Figure 2) indicated significant differences (*p* < 0.05) in the intensity of present pain, mental quality of life and depression, with medium effect sizes. Significant differences (*p* < 0.001) were found for self-efficacy in symptoms management, self-efficacy in pain control, and in the total self-efficacy score (with medium size effects, 0.2 < d < 0.49), as well as sleep disturbances and quantity (with large effect sized, d > 0.8).

## 4. Discussion

Previous MBCT studies have highlighted its efficacy for the treatment of depression and anxiety, with its use for chronic pain being more recent. The scarce research fundamentally comes from the same research group, which adapted the program utilized in this project, and which were centered on the study of headaches, a diagnosis that is absent in the present research study. Thus, the comparison of the results with previous research studies should be made with caution, as the sample diagnoses are not equivalent.

The effectiveness of MBCT on the symptoms of depression has been widely studied, and its use is recommended as a co-adjuvant therapy for unipolar depression [43,44,45] and for depression symptoms specifically in chronic pain conditions [46]. The previous evidence is consistent and coherent with that found in the present research study, and it highlights the importance of psychological and emotional factors as modulators of the sensory response to pain, although it remains to be determined to what extent and direction these variables are influenced, which is why more research on the mediational processes of pain is necessary.

As opposed to previous studies [44,47], no significant differences were found for anxiety. Nevertheless, the studies cited evaluated this variable with other instruments and populations with other diagnoses, including clinical anxiety disorders. Lastly, systematic reviews indicated that mindfulness-based stress reduction (MBSR) was more suitable for anxiety symptoms and MBCT for depression symptoms [43,44].

The results with the largest effect sizes were the improvements in sleep disturbances and quantity. These results are in agreement with the previous research studies with insomnia patients [48,49]. Some authors [45] explained that the reduction in insomnia through MBCT was due to the reduction of worries (a specific component of anxiety) produced by the therapy, which were associated with sleep problems in patients with anxiety. Despite the interest elicited by this hypothesis, more research is needed to better discern the relationships between these variables. It would be especially convenient to measure the different components of anxiety such as rumination with specific instruments.

Regarding pain, we found a significant reduction in the intensity of present pain, but not in the pain in the prior two weeks, which is similar to previous studies with MBCT [46,50,51] that pointed reduction in the interference of pain with no reductions in the everyday pain reported. Having in mind that the reduction in pain is not the primary objective of mindfulness-based treatments, the results allow us to think that the improvement in pain variables may not be solely due to the decrease in intensity but due to the improvement of other cognitive processes, like self-efficacy. In fact, in our study we found significant differences in self-efficacy for the total scale, symptom management, and pain control but not in the physical functioning subscale, consistent with previous studies [50,52]. Something similar happens with regard to the quality-of-life scales, since only the mental quality of life subscale improves, but not the physical one, partially coinciding with previous results [53,54]. Taking into account these results in conjunction with the previous evidence of these variables in MBCT, it is plausible to think that participants improved in the sense of control and coping, and consequently pain interference and mental quality of life, despite the fact that their baseline pain experience remained similar throughout treatment.

Concerning acceptance, an improvement in this variable could also be expected, parallel to the decrease in pain interference, as reported in a previous study [50] where they hypothesize the mediating capacity of acceptance in pain interference. However, we did not find this improvement in our study. It is risky to describe solid conclusions with these mixed results, having in mind the scarcity of the studies that have addressed this specific subject. Accordingly, significant differences were not found in the mindfulness skills when the treatment ended, in agreement with other previous studies with headaches [50,55], and in disagreement with other research studies, where differences were found in the short [47] and long term (6 months) [56]. However, there is a possibility that the effects on the mindfulness skills found in the present study may be long-term, for example some authors have found [56] improvements in mindfulness skills 6 months after training, with patients who had coronary disease and depression. In any case, it is curious to verify that the variables to which mindfulness-based interventions are directed are those that have been least affected by the treatment. A review [17] pointed out that the effect of the treatment on these skills is small and heterogeneous throughout the various studies found. Thus, this strengthens the need to continue conducting research on these variables and to study the role of the mindfulness-based interventions on mindfulness skills, acceptance of pain and its relationship with pain interference.

## 5. Conclusions

The MBCT was efficient for the reduction of sleep disturbances, intensity of present pain, and depression, and for the improvement of self-efficacy, mental quality of life, and the quantity of sleep. These results mostly coincide with the scarce previous studies, with the main limitation being the lack of a control group and long-term follow-up. The review of previous studies indicates that in some of the variables studied, statistical significance was found in the follow-up evaluations, despite not having shown significant changes in the post-test, which allows us to hypothesize that some variables that did not undergo changes in our study (such as mindfulness skills and acceptance of pain) could show significant differences in the long term. At the same time, it would allow us to check if those that underwent changes were maintained over time. Furthermore, it would have been interesting to compare results between participants who finished therapy and those who dropped out. Despite these limitations, the fact that this was a multi-center study performed by six professionals-in-training without any specific experience with MBCT (although they had experience with group therapy) and that a wide variety of pain-related diagnoses have been included (as it is representative of the samples of pain units in public hospitals) provides the results with a greater external validity and demonstrates the usefulness of this approach in transdiagnostic scenarios. Likewise, in the context of scientific literature, this is one of the few MBCT studies related to chronic pain, which was not from the same research group or about oncological pain.

## Figures and Tables

**Table 1:** Session contents.

**1. Abandoning the automatic habits of pain**
Introduction to the program: welcome, presentation of the participants. Establishment of rules, objectives, roles, and responsibilities. Theory of the door and introduction to the most common meditation practices.
**2. Facing the challenges**
Connections between thoughts–emotions–behaviors. Introduction of the ABC model. Stress-pain thermometer. Mindfulness with pleasurable experiences. Meditation centered on breathing.
**3. Breathing as the anchor**
Meditation based on the senses. Breathing as the anchor and sitting meditation. Working on unpleasant sensations. Awareness of stressing situations. Review of doubts and difficulties.
**4. Learning how to be present**
Sitting meditation: mindfulness of sounds and thoughts. Diary of stressful experiences and discussion about useless mental habits. Responsive 3-min breathing meditation. Movement-based mindfulness.
**5. Active acceptance**
Meditation in silence. The process of active acceptance. Automatic thoughts, intermediate beliefs, and main beliefs. Awareness of mental patterns. Sitting meditation.
**6. Thoughts as only thoughts**
Tendency towards interpretation. Seeing thoughts as only thoughts. Sitting meditation: working on difficulties. Relationship between emotional and physical state and thoughts. Tool for changing the point of view. Thermometer of pain. Mindfulness maintenance plan.
**7. Caring for oneself**
Sitting meditation: working with difficult thoughts, training on acceptance without judgement. Identification of warning signs and plans to decrease stress. Full attention to daily-life activities. Exchange of ideas about informal practices.
**8. Maintenance in the management of chronic pain**
Body scanner. Identification of red flags of stress and pain and use of options to face them. Experience of participants in the program. Metaphor of “mindfulness backpack” about the tools learned and how to continue using them. Maintenance plan. Meditation of the shell.

**Table 2:** Sociodemographic characteristic and medical diagnoses of the participants.

Variables	*N* (%)
Sex	
Male	13 (22.8)
Female	44 (77.2)
Marital status	
Single	10 (17.6)
Married	33 (57.9)
Divorced	8 (14)
Widowed	6 (10.5)
Employment status	
Active	11 (19.3)
On leave	9 (15.8)
Disability	10 (17.5)
Retired	15 (26.3)
Homemaker	12 (21.1)
Diagnosis	
Low back pain/lumbosciatic pain	22 (38.6)
Neck pain/cervicobrachialgia	7 (12.3)
Fibromyalgia	7 (12.3)
Rheumatic arthritis	6 (10.5)
Other medical conditions that imply chronic pain	15 (26.3)
Center	
General University Hospital of Alicante	23 (40.4)
Marina Baixa Hospital	11 (19.3)
Vega Baja Hospital	23 (40.4)

**Table 3:** *t*-test and Cohen’s d results.

	Pre	Post				
	M (DT)	M(DT)	t	gl	p	d
Intensity of pain last 3 days	7.54 (1.56)	8.17 (12)	−0.39	54	0.702	−0.073
Present pain intensity	2.95 (.65)	2.62 (0.73)	2.96	54	0.004	0.477
QL ^1^ physical	28.60 (5.62)	28.54 (2.31)	0.05	47	0.958	0.013
QL ^1^ mental	32.49 (12.44)	38.96 (13.26)	−2.98	47	0.005	−0.503
SE ^2^ symptoms	32 (12.71)	40 (17.96)	−4.01	56	0.000	−0.527
SE ^2^ physical	25.21 (13.91)	29 (14.7)	−1.93	56	0.059	−0.264
SE ^2^ pain control	11.98 (9.64)	18.14 (12.04)	−4.26	56	0.000	−0.564
SE ^2^ total	69.19 (31.54)	87.47 (41.11)	−3.78	56	0.000	−0.498
Anxiety	10.94 (4.06)	10.08 (4.11)	1.78	56	0.079	0.21
Depression	11.15 (4.80)	9.36 (4.79)	3.12	56	0.003	0.378
Sleep disturbances	30.95 (15.61)	58.83 (25)	−8.12	56	0.000	−10.337
Quantity of sleep	5.38 (1.44)	5.28 (1.36)	−8.12	56	0.000	0.071
Optimum sleep	0.07 (0.26)	0.05 (0.23)	0.444	52	0.659	0.081
Snoring	58.14 (41.53)	52.22 (36.94)	1.19	53	0.236	0.150
Awake	46.07 (34.09)	45.35 (31.33)	0.21	55	0.839	0.021
Somnolence	46.90 (24.78)	45.38 (23.10)	0.54	56	0.59	0.063
Suitability	30.70 (29.32)	30 (25.14)	0.19	56	0.849	0.021
Interference sleep 6 items	59.76 (21.64)	57.42 (21.14)	0.83	56	0.406	0.109
Interference sleep 9 items	60.25 (21.28)	56.16 (21.05)	10.42	56	0.160	0.193
Acceptance	38.33 (16)	40.28 (17.45)	−0.84	56	0.400	−0.116
Engagement in activities	24.64 (12.45)	24.47 (13.48)	0.1	56	0.921	0.013
Pain Willingness	13.68 (8.79)	15.80 (10.29)	−1.29	56	0.199	−0.221
Mindfulness attitude	3.85 (3.47)	3.72 (1.19)	0.28	55	0.781	0.05

^1^ QL = quality of life; ^2^ SE = self-efficacy.

## Data Availability

The data presented in this study are available on request from the corresponding author. The data are not publicly available due there are unpublished results.
